# Transcription Profiling of Rice Panicle in Response to Crude Toxin Extract of *Ustilaginoidea virens*

**DOI:** 10.3389/fmicb.2022.701489

**Published:** 2022-05-12

**Authors:** Rongtao Fu, Cheng Chen, Jian Wang, Yao Liu, Liyu Zhao, Daihua Lu

**Affiliations:** ^1^Institute of Plant Protection, Sichuan Academy of Agricultural Sciences, Chengdu, China; ^2^Key Laboratory of Integrated Pest Management on Crops in Southwest, Ministry of Agriculture, Chengdu, China; ^3^Crop Research Institute, Sichuan Academy of Agricultural Sciences, Chengdu, China

**Keywords:** rice, *Ustilaginoidea virens*, sugar transporter, differential expression gene, transcriptome, toxin, programmed cell death

## Abstract

*Ustilaginoidea virens* infects rice, causing rice false smut disease and reduced yields. During its growth, *U. virens* can also produce some toxins but less is known about the response mechanisms of the plant to *U. virens* toxins. *U. virens* toxins can inhibit the accumulation of total sugar in rice panicles. We used RNA sequencing to analyze the differential expression profile induced by infiltrating crude toxins into early growth-stage rice panicles. We compared the transcriptomes of the control and crude toxin-treated rice panicles and determined variable transcriptional responses under the action of the crude toxins. A total of 6,127 differentially expressed genes (DEGs) were identified. Among these genes, 3,150 were upregulated and 2,977 were downregulated. Gene Ontology (GO) and metabolic pathway enrichment analyses indicated that *U. virens* toxins mainly influenced glycometabolism, amino acid metabolism, and secondary metabolism of rice panicles. DEG analysis showed that the gene expression levels of 10 transcription factor families were significantly changed. Genes involved in phenylpropanoid biosynthesis, flavonoid biosynthesis, sugar transporters, and starch synthesis-related were significantly downregulated, including cytochrome P450, beta-glucosidase, CHS1, sucrose transporters, SWEETs, starch-branching enzymes, and UDP-glucose pyrophosphorylase. However, genes involved in programmed cell death (PCD) were significantly upregulated and contained cytochrome c, metacaspase, and protein kinase genes. The results indicate that *U. virens* toxins may act as the pathogenic factors to reduce stress resistance, disrupt total sugar accumulation and starch formation, and induce PCD.

## Introduction

*Ustilaginoidea virens* (Cook) Takahashi causes rice false smut (RFS), an epidemic disease that causes serious rice yield losses (Ladhalakshmi et al., 2012; Sun et al., 2013). *U. virens* infects rice floral organs at the rice booting stage and forms false smut balls. The mature orange-yellow balls eventually break and release powdery yellow spores (Tang et al., 2013; Hu et al., 2014). The released spores cause secondary infections in the rice fields. RFS has been largely ignored because it formerly occurred only sporadically in most rice-growing areas. However, the disease is now a more serious problem due to large-scale planting of high-yield rice cultivars and hybrids, heavy applications of N fertilizer, and global climate change. In China, the incidence rate of RFS has increased greatly, and it now affects more than 30% of the rice-planting area in some years (Guo et al., 2012; Lu et al., 2018).

In addition to the yield losses caused by RFS, *U. virens* produces large quantities of toxins that contaminate rice grains and straw and are toxic to humans and livestock (Ludueña et al., 1994; Li et al., 1995; Shan et al., 2013). Previous studies reported that *U. virens* produce three types of toxins, namely, ustiloxins (13-membered-ring cyclopeptides), ustilaginoidins (9, 9′-linked bis-naphtho-γ-pyrones), and sorbicillinoids (Sun et al., 2017; Wang et al., 2017; Meng et al., 2019). Ustiloxins and ustilaginoidins have cytotoxicites and phytotoxicities to suppress the growth of human tumor cell and inhibit the elongation of rice radicle and germ (Koyama et al., 1988; Sun et al., 2017, 2020). Ustiloxins are the most abundant toxins detected in false smut balls and culture fluid of *U. virens* mycelia (Chen et al., 2004; Wang et al., 2017).

Toxins are a crucial pathogenic factor of phytopathic fungi that affect the subcellular and biological processes of the host (Wang et al., 2019). Solanapyrone A produced by *Alternaria solani* can inhibit DNA polymerase in host cells, affecting host gene expression (Mizushina et al., 2002). Deoxynivalenol (DON) can act on the cell wall, chloroplast, plasmalemma, and endoplasmic reticulum in host cells, and play a crucial role in infection, pathogenesis, and colonization for *Fusarium gramineus* on wheat spikes (Kang et al., 2004; Wang et al., 2020). Elsinochrome (ESC) produced by *Elsinoe arachidis* is a photosensitizing perylenequinone toxin, which leads to an increase in peroxidation of endoplasmic membrane of host, aggravates membrane damage, and kills cells by producing a large amount of reactive oxygen species (ROS) (Jiao et al., 2019). Because of the significance of toxins in the pathogenic process of pathogens, understanding the molecular mechanisms of toxin action on plant host is of utmost importance.

A transcriptome represents a comprehensive set of loci transcribed from the genome. It provides insights into expression patterns, functional elements, and regulation of regions of the genome transcribed under different conditions. Transcriptional dynamic analysis of host plants revealed that the WRKY and v-myb avian myeloblastosis viral oncogene homolog (MYB) transcription factors, abscisic acid (ABA) and Ca^2+^, respectively, play the critical roles in signal transduction during the rice– *U. virens* interaction (Chao et al., 2014). In addition, differential expression profiling, by transcriptome analysis, of the early response to *U. virens* between false smut-resistant and susceptible rice varieties indicated that WRKY transcription factors, PR proteins, and diterpene phytoalexins were important in rice resistance to RFS (Han et al., 2015). Transcriptome analyses were conducted for other plant–pathogen interactions by RNA sequencing (RNA-seq), including rice and *Rhizoctonia solani* (Zhang et al., 2017), rice and *Magnaporthe oryzae* (Bagnaresi et al., 2012), maize and *Sporisorium reilianum* (Zhang et al., 2013), and wheat and *Fusarium graminearum* (Xiao et al., 2013).

RNA sequencing has been used to study differential expression profiling of rice seedling root response to *U. virens* mycotoxins (Wu et al., 2018) and the effect of ustiloxin A on the gene expression of rice spikelets (Hu et al., 2020). In this study, we performed a comparative transcriptome analysis of rice panicle response to *U. virens* toxins at 24 and 48 h after exposure. The results indicate that *U. virens* toxins may suppress the stress resistance, disrupt total sugar accumulation and starch formation of rice, and induce PCD in rice panicles. The toxins accomplish this by inhibiting the gene expression of cytochrome P450, beta-glucosidase, CHS1, sucrose transporters (SUTs), SWEETs, starch-branching enzymes (SBEs), and UDP-glucose pyrophosphorylase (UGPase).

## Materials and Methods

### Plant Materials

*Oryza sativa* L. spp. *indica* cultivar 93-11 was used in this study. Rice plants were grown in an air-conditioned greenhouse with the temperatures ranging from 20°C at night to a maximum of 36°C during the day. After treatment, rice plants were maintained at 25/30°C (night/day), covered with an inner solar-shade screen, and automatically sprayed water every 2 h for 10 min to maintain a relative humidity (RH) > 85%. After 4 days, the inner solar-shade screen was closed, and the plants were grown under the normal greenhouse conditions at 25–35°C and 80–100% RH.

### Preparation of Crude Toxin Extracts of *U. virens*

The crude toxins of *U. virens* were prepared using the methods of Nakamura et al. (1994) and Chen et al. (2004), with some modifications. Briefly, mycelia of *U. virens* were cultured in potato sucrose (PS) fluid medium (in which PS was made from a boiled extract of 300 g of peeled potatoes and 20 g of sucrose) at 28°C under 120 rpm by shaking for 14 days. The liquid medium was collected and then concentrated to dryness under a vacuum at 60°C by a rotary evaporator. The dry matter was dissolved in the same volume of methanol as the liquid, shaken for 2 h to fully extract compounds, and then centrifuged. The supernatant was concentrated under the vacuum at 60°C by a rotary evaporator to a concentrated dry extract. The dry extract was stored at 4°C until needed. The crude toxin yield was approximately 2 mg from 100 ml of the liquid culture medium.

### Analysis of Crude Toxin Components

The component analysis of the crude toxins by high-performance liquid chromatography (HPLC) was carried out as described previously (Yin et al., 2012; Shan et al., 2013). The crude toxins were diluted with water, filtered using a 0.45-μm microporous membrane filter, and analyzed by HPLC Agilent 7890B (Agilent Technologies, CA, United States). Chromatographic separations were performed at 40°C using an Eclipse XDB-C18 column (250 mm × 4.6 mm, 5 μm). The mobile phase, composed of methanol–water– methanoic acid (400:70:1, V/V), was eluted at a flow rate of 0.8 ml/min, with UV detection at 220 nm. Mass spectrometry (MS) was performed using an Agilent 6410 triple quadrupole analyzer (Agilent Technologies, CA, United States) equipped with an electrospray ionization (ESI) source in positive ion mode. The ESI conditions were as follows: ion source, ESI positive; nebulizer gas, N_2_; nebulizer pressure, 45 psi; drying gas temperature, 350°C; drying gas flow rate, 10.0 L/min; capillary voltage, 4,000 V; and SCAN (scan beginning at 100 m/z and ending at 800 m/z) (Shan et al., 2012; Yin et al., 2012).

### Crude Toxin Treatment

To prepare a crude toxin inoculation solution, 2 mg of crude toxin was dissolved in 100 ddH_2_O to obtain a 0.02 mg/ml solution of crude toxins. Approximately 2 ml of crude toxin solution was injected into each rice panicle at the seventh stage of panicle development (Ding and Xu, 1959). The controls were injected with distilled water. The crude toxin-treated (CL) and control panicles (CK) were collected and immediately frozen in liquid nitrogen for RNA extraction and total sugar extraction. We chose two time points (24 h and 48 h) for RNA-seq and five time points (24, 48, 72, 96, and 120 h) for total sugar detection. There were three biological replicates for each sample. Other crude toxin-treated panicles were observed for disease symptoms after 20 days of growth.

### Determination of Total Sugar Content

The samples were prepared and processed using the plant total sugar determination kit (Solarbio, Beijing, China) according to the manufacturer’s instructions. The optical density (OD) values of the treated sample were measured with a Synergy H1 microplate reader (BioTek, Winooski, VT, United States) at 540 nm. The total sugar content was calculated by standard curve.

### RNA Extraction and Transcriptome Sequencing

Total RNA was prepared using TRIzol (Aidlab Biotechnologies, Beijing) according to the manufacturer’s protocol. The quality and quantity of isolated RNA were evaluated using 1% agarose gel and an Agilent 2100 Bioanalyzer (Agilent Technologies, Santa Clara, CA, United States). RNA with an RNA integrated number (RIN) value greater than 7.0 was used for transcriptome library production. Library construction and sequencing were performed at Sangon Biotech (Shanghai, China). Briefly, the mRNA was separated into shorter fragments (about 200 bp) using a fragmentation buffer and reverse-transcribed into complementary DNA (cDNA) with random primers. DNA polymerase I, RNase H, deoxyribonucleotide triphosphate (dNTP), and buffer were added to synthesize the second-strand cDNAs. Then, cDNA fragments were purified with a QiaQuick PCR extraction kit (Qiagen, Germany), washed with tris-ethylene diamine tetraacetic acid (TE buffer) for end repair and single nucleotide A (adenine) addition, and ligated to Illumina sequencing adapters. The ligation products were size-selected by agarose gel electrophoresis, enriched by PCR amplification, and sequenced using Illumina HiSeq™ 2,500 (Illumina, San Diego, CA, United States) (Lu et al., 2017).

### Analysis of Differential Expression Genes

To obtain high-quality clean reads, raw reads were filtered to remove adaptor bases, reads containing more than 10% of unknown sequences (N), and low-quality sequences. The clean reads were mapped to the reference genome of *Oryza sativa L.* ssp. *indica* using HISAT2 (Kim et al., 2015). No more than two mismatches were allowed in the alignment for each read. The gene expression level was calculated by the software StringTie (Pertea et al., 2015) and normalized using the transcripts per million method. DEGs were identified by comparing gene expression levels between toxin-treated and control panicles with a log_2_ fold-change ≥ 1 and a false discovery rate (FDR) < 0.05 (Benjamini and Yekutieli, 2001).

### Functional Enrichment Analysis

Gene Ontology (GO) and Kyoto Encyclopedia of Genes and Genomes (KEGG) enrichment analyses were performed using the top GO and ClusterProfiler R package, respectively (Yu et al., 2012). GO terms and KEGG pathway enrichment analyses were performed to identify significantly enriched rice DEGs compared with the whole genome background. Pathways with a *Q*-value ≤ 0.05 were considered significantly enriched in DEGs.

### Validation of RNA-Seq Data by Quantitative Reverse Transcription PCR

To validate the DEG results, quantitative reverse transcription PCR (qRT-PCR) was conducted on 15 DEGs with ubiquitin (*OsUBI*) as the internal reference. The primer sets used for qRT-PCR were designed according to the individual gene sequences (Supplementary Table 1). The cDNA was generated by reverse transcription using the First-Strand cDNA Synthesis Kit (TransScript, Beijing, China), following the manufacturer’s protocol. The qRT-PCR experiments were conducted using a qTower3G Real-Time PCR System (Analytik Jena AG, Germany) according to the manufacturer’s protocol. PCR was performed in 20 μl of reaction volume, containing 2 μl of cDNA, 0.4 μl of each specific primer, 10 μl SYBR Premix Ex Taq™ (Takara, Dalian, China), and 0.4 μl ROX reference dye. Each reaction was performed three times. The relative gene expression levels were calculated using the 2^–ΔΔCT^ method (Livak and Schmittgen, 2001).

### Statistical Analysis

The data from this study are expressed as means ± SD of three replicates for each treatment. Statistical analysis was performed using a one-way analysis of variance (ANOVA) using SPSS 19.0 (IBM, Armonk, NY, United States). The least significant difference was used to test whether the ANOVA result between different treatment groups was significant at *p* < 0.05.

## Results

### Extraction and Active Component Analysis of Crude Toxins

**FIGURE 1:**
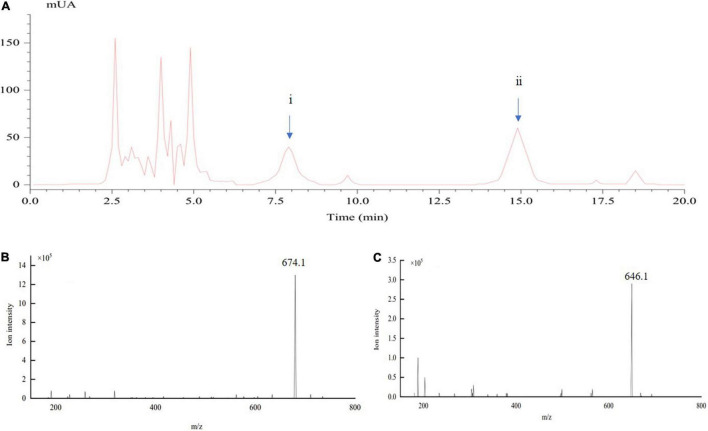
Determination of crude toxin compounds from the liquid culture of mycelia of *Ustilaginoidea virens* by LC-electrospray ionization (ESI)-MS. **(A)** represents the spectra of HPLC-UV; **(B,C)** show the ESI-MS spectra of ustiloxins **(A)** (peak ii) and **(B)** (peak i), respectively, appearing in **(A)**.

The crude toxins were extracted from the liquid culture of mycelia of *U. virens* (Nakamura et al., 1994; Chen et al., 2004). In this study, HPLC and MS were used for the active component analysis of crude toxins. Figure 1 shows the HPLC-UV and MS chromatograms of the crude toxins in 20 min. The ustiloxins were preliminarily identified by comparison of their retention times with the previous studies (Miyazaki et al., 2009; Shan et al., 2013). Retention time of the two ustiloxins was approximately 7.9 and 14.95 min (Figure 1A). They were further analyzed by ESI-MS, and the results are shown in Figures 1B,C. Based on the molecular weights of the ustiloxins previously reported (Koiso et al., 1994; Shan et al., 2013), 674.1 *m/z* (Figure 1B) should be the peak of the [M + H]^+^ ion of ustiloxin A, which corresponds to peak ii in Figure 1A. Similarly, 646.1 *m/z* (Figure 1C) should be the peak of the [M + H]^+^ ion of ustiloxin B, which corresponds to peak i in Figure 1A. Other peaks in Figure 1 were speculated to be other toxins according to the previous studies (Koiso et al., 1994; Shan et al., 2012; Wang et al., 2017; Meng et al., 2019).

### Symptoms and Total Sugar Content of Rice Spikelets After Treatment

**FIGURE 2:**
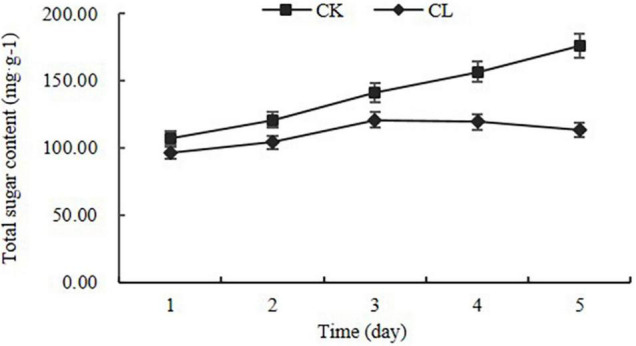
Changes in total sugar content in the crude toxin treatment (CL) and the control (CK). Error bars represent the standard errors for three replicates of total sugar content.

To confirm the biological activity of crude toxins on panicles, crude toxins were used to infiltrate the panicles at the seventh stage of panicle development. The panicle length of the CL group was shorter than that of the CK group (Supplementary Table 2). The color of the CL-spikelet shell became pale yellow, whereas the CK seed shell was light green. At the end, the CL-spikelet did not form full seeds (Supplementary Figure 1). In addition, the total sugar content of CL-spikelet was lower than that of the control. The total sugar content of the CL-treated spikelets decreased slowly on the 3rd day after treatment, while that of the CK increased continuously (Figure 2). These results indicate that crude toxins can inhibit the growth and development of the rice panicles and the accumulation of total sugar, and treated panicles were unable to accumulate rich starch to form full seeds.

### RNA-Seq Results of Transcriptome Samples

Illumina RNA-seq analysis of 12 samples yielded 68.81 Gbp of data and 277,405,639 reads (clean data) (Supplementary Table 3). From 93 to 96% of the clean reads were successfully aligned to the reference genome. We used sample-to-sample correlation analysis for the data analysis. The overall relatedness of the transcriptome at different times and different treatments was determined by clustering, and a sample correlation heatmap was generated for all samples (Supplementary Figure 2). For all samples, the three biological replicates showed good correlation, and the transcriptome data were closely related at each time point. These results indicate that the gene transcript data were reliable and suitable for further transcriptome analysis.

### Differential Gene Analysis of Rice Panicles

**FIGURE 3:**
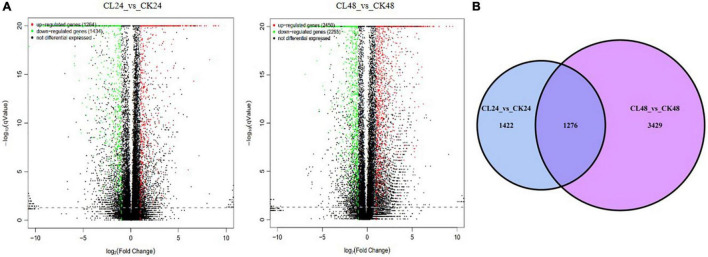
The DEGs between CL24 vs. CK24 and CL48 vs. CK48. Panel **(A)** represents volcano plot of DEGs between CL24 vs. CK24 and CL48 vs. CK48. Splashes represent different genes, where black indicates the mean number of genes without significant differential expression, red indicates the mean of significantly upregulated genes, and green indicates the mean of significantly downregulated genes. **(B)** Venn diagram showing the overlap of expressed genes between CL24 vs. CK24 and CL48 vs. CK48. Blue represents CL24 vs. CK24, and purple represents CL48 vs. CK48.

To determine which genes exhibit expression changes and the stage at which these changes occur, comparisons between the CL treatments, with the CK, were performed. Expressed genes with a log_2_ fold-change ≥ 1 and an FDR < 0.05 were designated as differentially expressed genes (DEGs). At 24 and 48 h, 2,698 DEGs (1,264 upregulated, 1,434 downregulated) and 4,705 DEGs (2,450 upregulated, 2,255 downregulated) were identified in the CL vs. CK, respectively (Figure 3A). A Venn diagram shows the DEGs that were common to two different time points, or specific to either time point in response to the toxin treatment (Figure 3B). Among them, 1,276 were identified as DEGs common to 24 and 48 h, 1,422 were categorized as DEGs only at 24 h, and 3,429 were identified as DEGs only at 48 h. Therefore, 6,127 DEGs were identified at the two time points (Supplementary Table 4). Among these genes, 3,150 genes were upregulated and 2,977 were downregulated (Table 1).

**TABLE 1:** Statistics of differentially expressed genes.

DEG set name	Up-regulated	Down-regulated	All DEGs
CK24_vs_CL24	1264	1434	2698
CK48_vs_CL48	2450	2255	4705
CK_vs_CL	3150	2977	6127

### Gene Ontology Enrichment Analysis

**FIGURE 4:**
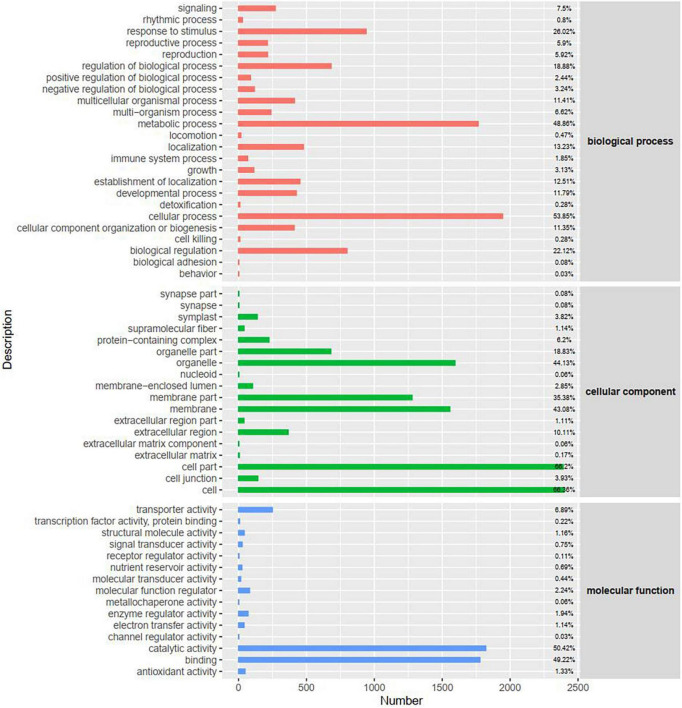
Gene Ontology (GO) classification of DEGs at 48 h. The *x*-axis represents the number of DEGs.

To investigate the functional distribution of unigenes, we used Gene Ontology (GO) annotations to classify the enriched DEGs between the control and fungal toxin treatment groups (Figure 4 and Supplementary Figure 3). All the unigenes annotated in the GO database were classified into three major categories (biological processes, cellular components, and molecular functions). Within biological processes, cellular process, metabolic process, response to stimulus, and biological regulation were the major common GO terms at 24 and 48 h. Under the cellular component category, most of the unigenes were enriched in cell, cell part, organelle, membrane, and membrane part. For the molecular function category, unigenes were mostly involved in binding activity, catalytic activity, and transporter activity.

### Analysis of Metabolic Pathways

To further investigate functional classifications and pathway assignments of rice panicle genes after CL treatment, all DEGs were analyzed against the Kyoto Encyclopedia of Genes and Genomes (KEGG) database. Approximately 5,203 DEGs were successfully annotated and assigned to 258 pathways. Among them, 41 pathways were significantly enriched and categorized in five groups, including cellular processes, genetic information processing, environmental information processing, metabolism, and organismal systems. The top 20 pathways with the most abundant DEGs at the two time points are shown in Table 2. At 24 and 48 h, the primary pathways were “starch and sucrose metabolism,” “phenylpropanoid biosynthesis,” “Carbon metabolism,” “Plant hormone signal transduction,” and “biosynthesis of amino acids.” Therefore, the data showed that the CL treatment had a significant effect on glucose metabolism, amino acid metabolism, and secondary metabolism. The KEGG pathway annotations provided a valuable resource for investigating the specific processes, functions, and pathways involved in rice spikelet response to the crude toxins.

**TABLE 2:** The top 20 enriched KEGG pathways for DEGs in 24 h and 48 h.

Pathway ID	Pathway	24 h		48 h	
			
		Number of DEGs	*Q*-value	Number of DEGs	*Q*-value
ko00500	Starch and sucrose metabolism	41	4.83E-08	68	3.21E-07
ko01200	Carbon metabolism	41	5.46E-08	49	3.29E-07
ko00010	Glycolysis/Gluconeogenesis	29	6.14E-08	31	3.53E-07
ko00040	Pentose and glucuronate interconversions	26	6.47E-08	27	3.56E-07
ko00620	Pyruvate metabolism	12	7.28E-08	23	3.71E-07
ko04144	Carbon fixation in photosynthetic organisms	19	6.62E-08	27	3.63E-07
ko00520	Amino sugar and nucleotide sugar metabolism	24	5.49E-08	44	3.30E-07
ko01230	Biosynthesis of amino acids	29	5.51E-08	45	3.37E-07
ko00480	Glutathione metabolism	14	7.36E-08	21	3.73E-07
ko00270	Cysteine and methionine metabolism	18	7.31E-08	23	3.71E-07
ko04141	Protein processing in endoplasmic reticulum	18	7.94E-08	21	3.73E-07
ko00230	Purine metabolism	15	9.68E-08	18	4.32E-07
ko01212	Flavonoid biosynthesis	11	9.38E-08	18	3.90E-07
ko00190	Oxidative phosphorylation	10	8.73E-08	20	3.78E-07
ko04075	Plant hormone signal transduction	40	4.84E-08	61	3.21E-07
ko00940	Phenylpropanoid biosynthesis	41	5.28E-08	54	3.25E-07
ko04626	Plant–pathogen interaction	25	6.04E-08	41	3.50E-07
ko04016	MAPK signaling pathway – plant	24	6.24E-08	28	3.54E-07
ko04910	Insulin signaling pathway	20	8.62E-08	20	3.76E-07
ko04721	Synaptic vesicle cycle	13	9.38E-08	18	3.90E-07

**FIGURE 5:**
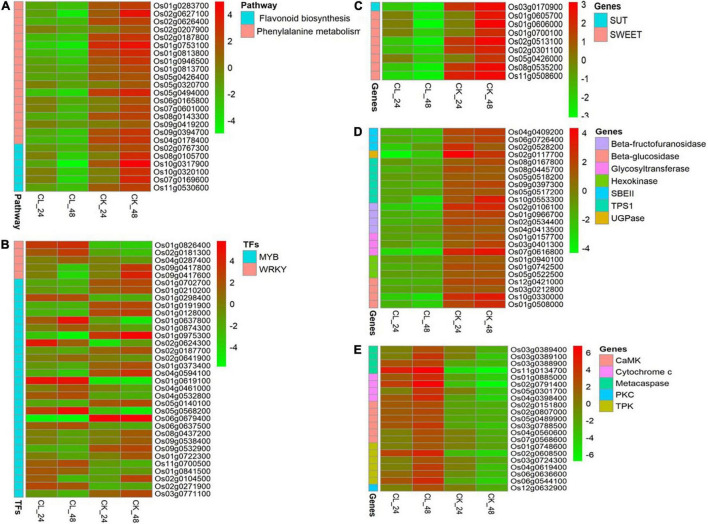
Expression pattern of differentially regulated genes in CL and CK at 24 and 48 h, respectively. **(A)** Phenylpropanoid biosynthesis and flavonoid biosynthesis pathways. **(B)** WRKY and v-myb avian myeloblastosis viral oncogene homolog (MYB) genes. **(C)** Sugar transporter genes. **(D)** Starch synthesis-related genes. **(E)** PCD-related regulatory genes.

Phenylalanine metabolism is an important secondary metabolic pathway in plants, which can regulate their stress tolerance (Zhang et al., 2020). To identify potential regulatory genes related to phenylalanine metabolism, we identified DEGs by comparing the expression changes between the CK and CL groups. Of the genes involved in phenylalanine metabolism that were expressed at 24 h, fewer were expressed at a lower level in the CL group than the CK group at 48 h. The difference between the CL and CK groups can be seen in the heatmap (Figure 5A and Supplementary Table 5). These genes included phenylalanine and histidine ammonia-lyase, cytochrome P450, beta-glucosidase, flavonol reductase/cinnamoyl-CoA reductase, and alcohol dehydrogenase. These results indicate that *U. virens* toxins may reduce the stress resistance of rice by downregulating the gene expression of some regulatory enzymes in phenylalanine metabolism in the young panicles.

We also analyzed the expression of related genes in the flavonoid biosynthesis pathway. A number of four cytochrome P450, one CHS1, and one iron/ascorbate family oxidoreductase genes were significantly downregulated at 48 h (Figure 5A and Supplementary Table 5).

### Effects of Crude Toxins on Plant Transcription Factors

Transcription factors (TFs) are essential for the regulation of plant gene expression. TFs are widely involved in the regulation of plant growth and development, environmental stress responses, and plant morphogenesis (Jiang et al., 2012). In this study, DEG analysis showed that the expression levels of 71 (29 upregulated, 42 downregulated) and 103 (46 upregulated, 57 downregulated) plant TFs were changed at 24 and 48 h, respectively. These DEGs belonged to 10 TF families, including MYB, WRKY, MADS-box, GATA, HOX, multiprotein bridging factor 1 (MBF1), heat shock transcription factor (HSF), basic helix–loop–helix (bHLH), GT-2, and X-box binding protein (XBP-1) (Table 3 and Supplementary Table 6). All of these TFs participated in plant regulation of abiotic stress, except HSF. The other nine TFs were involved in plant growth and development regulation, and only four others were involved in plant regulation of biological stress.

**TABLE 3:** Plant transcription factors affected by crude toxins produced *U. virens.*

Number of affected transcription factors (TFs)							
	
	24 h			48 h			
	
TFs family	Up-regulated	Down-regulated	Total	Up-regulated	Down-regulated	Total	Main regulatory functions in plants
MYB	11	12	23	12	17	29	a,b,c
WRKY	2	0	2	3	2	5	a,b,c
MADS-box	1	5	6	2	7	9	a,c
GATA	1	4	5	2	7	9	a,c
HOX	5	9	14	12	10	22	a,c
MBF1	2	0	2	2	0	2	a,b,c
HSF	2	6	8	4	8	12	b,c
bHLH	2	3	5	4	2	6	a,b,c
GT-2	2	3	5	2	4	6	a,c
XBP-1	1	0	1	3	0	3	a,c
Total	29	42	71	46	57	103	

*a, regulation of plant growth and development.*

*b, plant resistance to biotic stresses.*

*c, plant tolerance to abiotic stresses.*

### Differential Expression of WRKY and v-myb Avian Myeloblastosis Viral Oncogene Homolog (MYB) Transcription Factors

The WRKY TFs are one of the largest families of transcriptional regulators in higher plants. They regulate the plant hormone signal transduction pathway and play the important roles in plant processes in response to biotic and abiotic stress (Jiang et al., 2017). We identified five WRKY genes that were differentially expressed in two different periods after fungal toxin treatment (Figure 5B and Supplementary Table 5). A number of two WRKY TFs were significantly downregulated, and three WRKY TFs were significantly upregulated at 48 h. Among them, *OsWRKY24* (Os01g0826400) and *OsWRKY71* (Os02g0181300) genes were induced between 24 and 48 h.

The MYB family proteins are one of the largest TF families in plants and they are involved in plant growth and development, stress response, product metabolism, and other processes (Ambawat et al., 2013). A total of 29 MYB genes in this study were activated two times (Figure 5B and Supplementary Table 5). Among them, 11 MYB genes were upregulated and 12 MYB genes were downregulated in both periods.

These findings indicate that some WRKY and MYB transcription factors may be involved in rice transcriptome regulation under the action of *U. virens* toxins.

### Sugar Transporter Gene Analysis

Sugar transporters play the important roles in many physiological processes such as sucrose distribution to plant tissues or organs, transport of other small molecules, grain filling, pollen development, and response to biotic and abiotic stresses (Williams et al., 2000). There are three main types of sugar transporters in plants, including sucrose transporters (SUTs), monosaccharide transporters (MSTs), and SWEETs (Williams et al., 2000; Yuan and Wang, 2013). We identified one SUT gene and eight SWEET genes that were significantly differentially expressed after fungal toxin treatment (Figure 5C and Supplementary Table 5). Among them, *OsSUT1* (Os03g0170900), *OsSWEET4* (Os02g0301100), *OsSWEET11* (Os01g0605700), *OsSWEET14* (Os11g0508600), and *OsSWEET15* (Os02g0513100) were significantly downregulated at both time points in CL. The SUTs and SWEETs are responsible for sucrose transport, regulating the starch synthesis and rice reproductive development (Chen et al., 2010; Li et al., 2011). Therefore, our results show that *U. virens* toxins may inhibit total sugar accumulation and the formation of full seeds by reducing the sucrose sugar transporters SWEET and SUT.

### Starch Synthesis-Related Genes in Rice Panicles

Under abiotic stress, various key enzymes of starch formation are inhibited and this can hinder starch accumulation in the grain (Thitisaksakul et al., 2012). We found that CL had no starch accumulation in grain which indicated that starch synthesis was damaged. This may have resulted from the effects of the *U. virens* toxins. Starch synthesis-related genes were downregulated in CL and those related to starch-branching enzyme II (SBE II) genes (Os04g0409200, Os06g0726400, and Os02g0528200) and UDP-glucose pyrophosphorylase (UGPase) gene (Os02g0117700). SBE and UGPase are essential for the synthesis of the starch granule and normal endosperm starch accumulation (Jeon et al., 2010). In addition, beta-fructofuranosidase, glycosyltransferase, beta-glucosidase, hexokinase, and trehalose-6-phosphate synthase component (TPS1) genes were also downregulated in CL. These results suggest that *U. virens* toxins may reduce starch accumulation by downregulating genes in the starch biosynthetic pathway (Figure 5D and Supplementary Table 5).

### Programmed Cell Death-Related Regulatory Gene Analysis

Programmed cell death is a physiological process occurring during the development and in pathological conditions of animals and plants (Jabs, 1999). It is a death process that cells actively strive for in order to adapt to stressful environments and involves a series of related factors such as cytochrome c (Cyt c), metacaspase, and protein kinase (Ding et al., 2008; Chichkova et al., 2010). Here, we identified four Cyt c genes, four metacaspase genes, and three types of protein kinase genes [calmodulin-dependent protein kinase (CaMK), tyrosine protein kinase (TPK), and protein kinase C (PKC)] that were differentially expressed in two different periods after fungal toxin treatment (Figure 5E and Supplementary Table 5). Of these, two metacaspase genes, three Cyt c genes, and six protein kinase genes were significantly upregulated at both time points in CL. According to the previous studies, Cyt c, metacaspase, and protein kinase play the key roles in the regulation of PCD in plants (Van Durme and Nowack, 2016). Therefore, our results show that *U. virens* toxins can activate the expression of PCD-related regulatory genes, induce the programmed death of rice panicles, and ultimately cause the failure of rice to form full seeds.

### Validation of Differentially Expressed Genes by Quantitative Quantitative Reverse Transcription PCR Analyses

**FIGURE 6:**
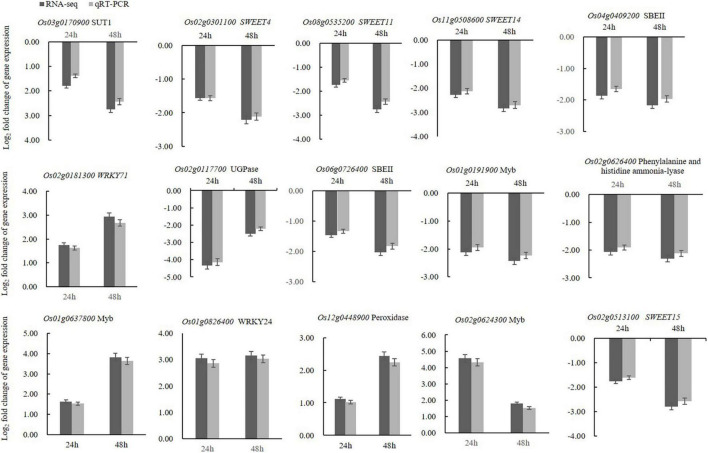
qRT-PCR analyses of fifteen selected DEGs confirmed that these genes were upregulated or downregulated in the crude toxin treatment (CL) at both 24 and 48 h. Log_2_ fold-change in CL transcripts with respect to the transcript levels in CK rice panicles is shown. Error bars represent the standard errors for three replicates of qRT-PCR assays.

To validate the DEGs obtained by transcriptome analysis, qRT-PCR was used to confirm the expression levels of 15 selected unigenes, with *OsUBI* serving as the reference gene (Supplementary Table 1). The rice sugar transport proteins such as *SUT1*, *SWEET4*, *SWEET11*, *SWEET14*, and *SWEET15* were selected for validation, as well as WAKY and MYB transcription factors. Several genes involved in metabolic pathways were also selected for validation. The qRT-PCR results showed that the expression patterns of the selected unigenes were consistent with the RNA-seq analysis (Figure 6). Therefore, the qRT-PCR results confirmed that the transcriptome analysis was reliable.

## Discussion

Plant pathogenic toxins are the toxic non-enzymatic compounds produced by plant pathogens, which often plays an important role in the process of pathogen colonization and is one of the main factors leading to plant diseases (Ismaiel and Papenbrock, 2015). Since the toxin of *Alternaria kikuchiana* was first reported (Tanaka, 1933), researchers began to study the toxins produced by many pathogenic fungi. Different plant pathogenic toxins can produce different phytotoxic effects and symptoms on plants. The phytotoxic effect of aflatoxin was observed based on the remarkable inhibitory effect on chlorophyll and carotenoid synthesis and reduction of seed germination and seedling growth of sesame (Samuel and Valentine, 2014). Tenuazonic acid can induce small brown necrotic spots on leaves and increase ROS production in plants (Aver’yanov et al., 2007). In this study, we found that *U. virens* toxins can suppress the growth and development of rice panicles and the accumulation of total sugar. This can prevent the accumulation of starch and the formation of full seeds. In addition to *U. virens*, other fungal diseases of rice, *M. oryzae* and *R. solani*, can also produce toxins and cause disease spots in rice leaves (Fujita et al., 1994; Vidhyasekaran et al., 1997). Understanding the *U. virens* toxins can provide a basis for determining the pathogenic mechanism of *U. virens* and help improve breeding for RFS resistance.

RNA sequencing is often used in transcriptome analyses to study the genome-wide expression profiling and regulation in plant hosts in response to pathogen infection. We used RNA-seq to identify genes differentially expressed between the control and plants treated with *U. virens* toxins. Comparative transcriptome analyses showed that the expression levels of 6,127 genes (3,150 upregulated and 2,977 downregulated) were significantly changed in rice panicles by treatment with *U. virens* toxins at 24 and 48 h postexposure. The changes in young panicle gene expression in this study were different from those reported by previous studies (Hu et al., 2020). This study shows that *U. virens* toxins can induce more changes in the gene expression of young panicles, which is a new observation. This is probably because we treated young panicles with crude toxins, which contain not only ustiloxin A but also ustiloxin B and other toxins.

The GO term analysis of DEGs showed that catalytic activity and transporter activity were the most enriched in the molecular function category. Based on the KEGG analysis, the most DEGs were annotated in “starch and sucrose metabolism,” “phenylpropanoid biosynthesis,” “Carbon metabolism,” “Plant hormone signal transduction,” and “biosynthesis of amino acids.” Previous studies reported that *U. virens* mycotoxins retarded the growth of the rice seedling roots mainly through disturbing respiration and amino acid metabolism (Wu et al., 2018). Rice panicles are nutrient accumulation organs, and so, “starch and sucrose metabolism” and “carbon metabolism” of rice panicles are mainly related to starch synthesis. Starch is the most important end product of cereal growth and development. However, many synthases are involved in the total process of starch synthesis (Thitisaksakul et al., 2012). Phenylpropanoids are plant secondary metabolites derived from phenylalanine. They have many functions, both as structural and as signaling molecules. It is an important pathway for the synthesis of lignin, phytoalexin, and phenol (Ouyang and Xue, 1988). Therefore, it is possible that *U. virens* toxins reduce starch accumulation, starch synthases, and rice stress resistance by disturbing phenylpropanoid biosynthesis.

Transcription factors are indispensable regulators in the life activities of higher plants. They occupy a large part of the plant genome and are important “switches” of downstream target genes (Ambawat et al., 2013). We found that *U. virens* toxins could activate changes in the expression levels of 10 families of TFs in rice panicles, including WRKY and MYB. The WRKY transcription factor superfamily in rice contains more than 100 members and is important in the regulation of defense responses (Pandey and Somssich, 2009). A total of five WRKY TFs were differentially regulated under the action of *U. virens* toxins. In particular, *WRKY24* and *WRKY71* were upregulated in CL at 48 h. Previously, *OsWRKY24* and *OsWRKY71* were reported to be involved in *M. oryzae* and *U. virens* attacks (Wang et al., 2014; Han et al., 2015), and *OsWRKY71* is also activated by *Xanthomonas oryzae* infection (Ryu et al., 2006). Overexpression of *OsWAKY71* can enhance resistance to *M. oryzae* and *X. oryzae* infection (Liu et al., 2007). MYB proteins are also the important transcription factors involved in the regulation of plant defenses and abiotic stress resistance (Ambawat et al., 2013). Several MYB TFs were activated in this study. Taken together, these findings demonstrate that some WRKY and MYB transcription factors are important in rice transcriptome regulation under the action of *U. virens* toxins.

Sugar transporters are a class of typical transmembrane binding proteins. They are involved in the transport and distribution of photosynthates in plants, which regulate physiological processes such as plant response to stress, resistance to pathogen infection, and seed formation and development (Frommer et al., 2013). There are three main types of sugar transporters in plants, including sucrose transporters (SUTs), monosaccharide transporters (MSTs), and SWEETs (Williams et al., 2000; Yuan and Wang, 2013). In this study, one *SUT* gene and eight *SWEET* genes were significantly downregulated at both time points after crude toxin treatment. Such responses may be different from the responses to *U. virens* infection, which possibly upregulates the expression of *SWEET* genes because of growth and smut ball formation (Chao et al., 2014; Fan et al., 2015). Previously, *SUT1* was shown to mediate sucrose transport from phloem to other tissues and organs in plants (Kühn et al., 2003), *OsSWEET4* is responsible for hexose transport and can promote starch storage in seed endosperm (Sosso et al., 2015). *OsSWEET11* and *OsSWEET14* are responsible for regulating starch synthesis (Chen et al., 2010), and *OsSWEET15* is involved in regulating rice reproductive development (Li et al., 2011). Therefore, our results suggest that *U. virens* toxins may inhibit the accumulation of total sugar and the formation of filled seeds by downregulating the expression levels of the sucrose transporters SWEET and SUT genes.

Starch synthesis in rice seeds is a complex process that involves a series of genes and gene families including SBEs, UDP-glucose pyrophosphorylase (UGPase), and starch synthases related to starch synthesis (Thitisaksakul et al., 2012). Abiotic stress can affect the synthesis and regulation of rice starch. SBEs are mainly responsible for the introduction of starch side chains in rice seed starch synthesis (Stitt and Zeeman, 2012). UGPase is essential for the synthesis of the starch granule and normal endosperm starch accumulation (Jeon et al., 2010). In this study, three SBE genes and one UGPase gene were identified to be significantly downregulated in crude toxin-treated panicles. Several other starch synthesis-related genes were also downregulated. These results suggest that *U. virens* toxins reduce starch accumulation to form full seeds by downregulating genes in the starch biosynthetic pathway.

Programmed cell death is an active mode of death regulated by genes at a specific stage of an organism’s growth and development, or in response to changes in the external environment (Jabs, 1999; Van Durme and Nowack, 2016). It has been reported that many factors play a crucial role in the regulation of PCD in plants, such as mitochondria, Cyt c, metacaspase, protein kinase, and Ca^2+^ (Chichkova et al., 2010; Gilroy et al., 2016). This study revealed that four Cyt c genes, four metacaspase genes, and 13 protein kinase genes were significantly upregulated in crude toxin-treated panicles. Previous studies have shown that Cyt c can be released into the cytoplasm from the mitochondria as an initiation factor to induce apoptosis during PCD (Balk et al., 1999). Metacaspase, which is a caspase-like protease, is an important initiator of PCD and can directly lead to the disintegration of apoptotic cells after activation (Sanmartín et al., 2005). Many protein kinases have been reported to be involved in the signal transduction of many kinds of apoptosis and to dramatically regulate, and sometimes accelerate, apoptosis, including CaMK, TPK, and PKC (Ding et al., 2008). Taken together, these findings suggest that *U. virens* toxins can induce the apoptosis of rice panicles by activating the expression of Cyt c, metacaspase, and protein kinase genes.

## Conclusion

The comparison of the expression profiles of the control and *U. virens* toxin-treated rice at two time periods following treatment revealed the molecular mechanisms of toxins on rice panicles. *U. virens* toxins activated multiple metabolic pathways, and the DEGs were mainly involved in glucose metabolism, amino acid metabolism, and secondary metabolism. A series of TFs, including WRKY and MYB, were induced by *U. virens* toxins. Phenylpropanoid biosynthesis, flavonoid biosynthesis, sugar transporters, and starch synthesis-related genes were significantly inhibited by *U. virens* toxins. Moreover, *U. virens* toxins can significantly upregulate PCD-related regulatory genes. This demonstrated that *U. virens* toxins can negatively affect the growth and development of rice panicles. Further research is needed to identify the toxin components and the molecular functions of a single toxin produced by *U. virens* in plants. These data increase our understanding of the molecular mechanism of *U. virens* toxins in rice.

## Data Availability Statement

The datasets presented in this study can be found in online repositories. The names of the repository/repositories and accession number(s) can be found below: https://www.ncbi.nlm.nih.gov/, PRJNA727620.

## Author Contributions

RF and DL were involved in designing the experiments. RF and LZ performed the experiments. RF, YL, JW, and CC helped to analyze the data and wrote the manuscript. All authors contributed to the article and approved the submitted version.

## Conflict of Interest

The authors declare that the research was conducted in the absence of any commercial or financial relationships that could be construed as a potential conflict of interest.

## Publisher’s Note

All claims expressed in this article are solely those of the authors and do not necessarily represent those of their affiliated organizations, or those of the publisher, the editors and the reviewers. Any product that may be evaluated in this article, or claim that may be made by its manufacturer, is not guaranteed or endorsed by the publisher.

## Supplementary Material

Supplementary Figure 1 Symptoms of panicles after crude toxin treatment. **(A)** Then, 5 days after crude toxin treatment; **(B)** 20 days after crude toxin treatment. CL, crude toxin-treated rice panicles; CK, the control.

Supplementary Figure 2 Clustering and heatmap of correlation analysis among all samples. The color block represents the correlation index value. The darker the green color, the lower the correlation among samples, and the darker the red color, the higher the correlation among samples.

Supplementary Figure 3 GO classification of DEGs at 24 h. The *x*-axis represents the number of DEGs.
