# Supplementary figures and images for: Transcription Profiling of Rice Panicle in Response to Crude Toxin Extract of Ustilaginoidea virens
Source: Front Microbiol. 2022 May 12;13:701489. doi: 10.3389/fmicb.2022.701489 (PMC9135463; doi:10.3389/fmicb.2022.701489)

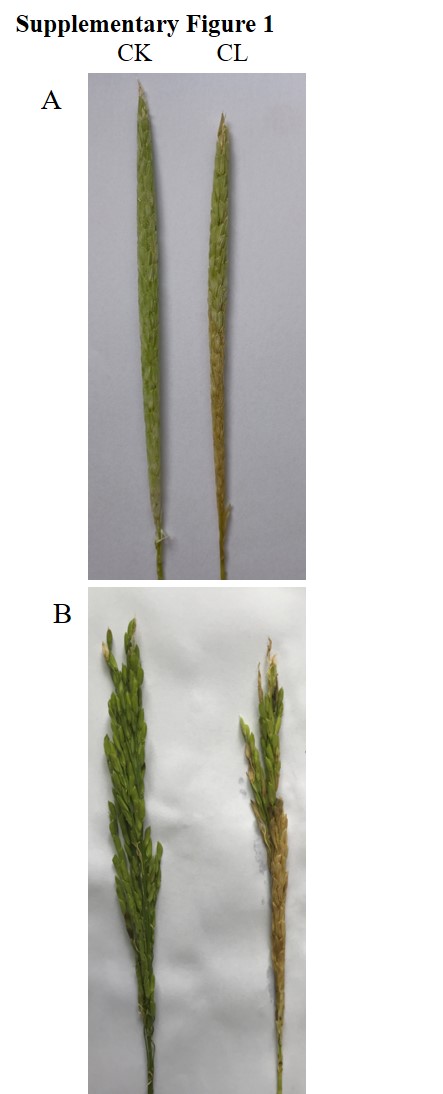

Supplement: Supplementary Figure 1 — Symptoms of panicles after crude toxin treatment. (A) Then, 5 days after crude toxin treatment; (B) 20 days after crude toxin treatment. CL, crude toxin-treated rice panicles; CK, the control. [file Image_1.jpg]

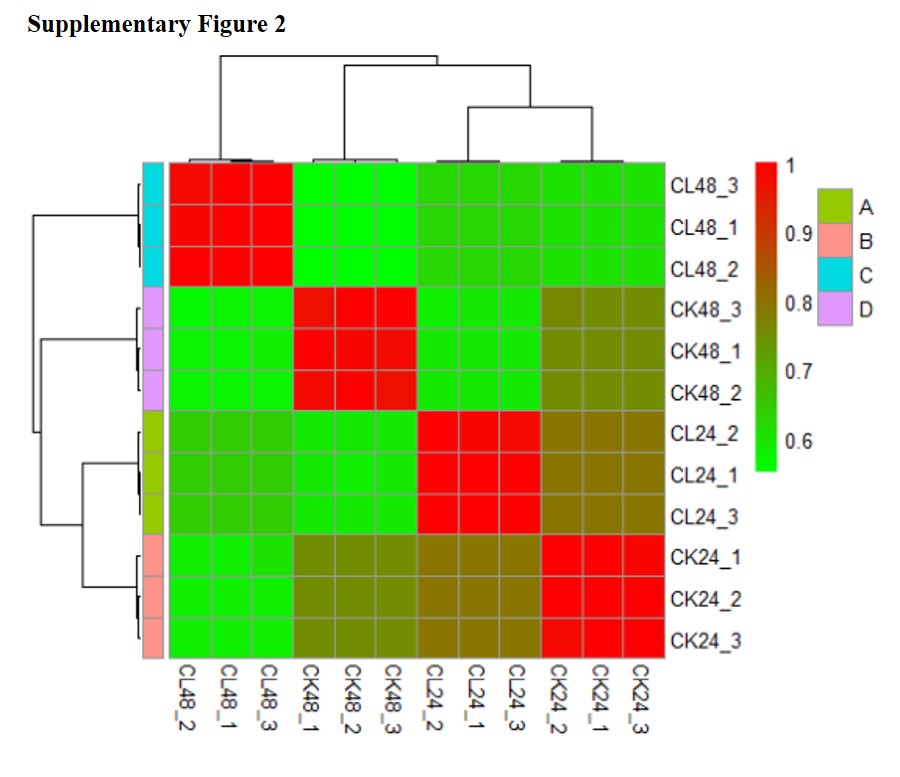

Supplement: Supplementary Figure 2 — Clustering and heatmap of correlation analysis among all samples. The color block represents the correlation index value. The darker the green color, the lower the correlation among samples, and the darker the red color, the higher the correlation among samples. [file Image_2.jpg]

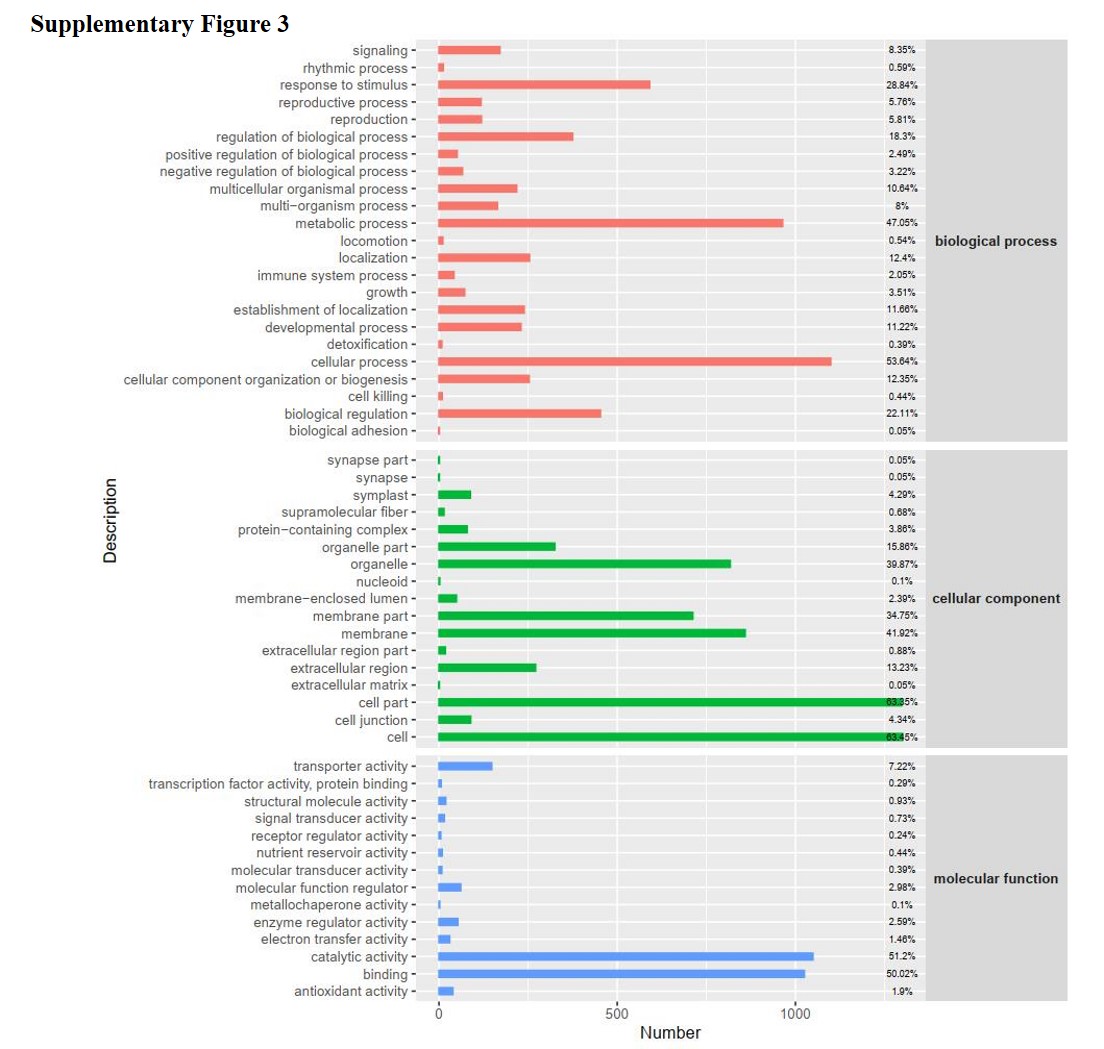

Supplement: Supplementary Figure 3 — GO classification of DEGs at 24 h. The x-axis represents the number of DEGs. [file Image_3.jpg]
